# Editorial: The role of microbial communities in environmental engineering systems

**DOI:** 10.3389/fmicb.2025.1644856

**Published:** 2025-07-10

**Authors:** Yuepeng Sun, Dunzhu Li, Bo Li, Keli Zhao

**Affiliations:** ^1^School of Ecology and Environment, Inner Mongolia University, Hohhot, China; ^2^Department of Civil, Structural and Environmental Engineering, Trinity College Dublin, Dublin, Ireland; ^3^School of Ecology, Environment and Resources, Guangdong University of Technology, Guangzhou, China; ^4^College of Environmental and Resources Sciences, College of Carbon Neutrality, Zhejiang A&F University, Hangzhou, China

**Keywords:** microbial communities, wastewater, sewage sludge, animal manure, food wastes

## Introduction

Microbial communities, comprising bacteria, archaea, fungi, algae, and viruses, exhibit remarkable ubiquity and broad applications across diverse fields. However, their systematic integration into environmental engineering systems remains relatively underexplored. Advances in microbial analysis technologies and methods (e.g., high-throughput sequencing, meta-omics, and bioinformatics) are enabling better integration of microbial research findings into the design and optimization of environmental engineering systems. Despite these advancements, significant gaps remain in our understanding of how microbial communities can be harnessed to improve the efficiency and effectiveness of waste treatment processes. Our Research Topic was initiated on 2 September 2024 and closed on 29 April 2025, with the help of handling editors from *Frontiers*, aiming to advance knowledge of the roles of microbial communities in diverse environmental engineering systems.

## Outline of contributions

Our Research Topic attracted a wide range of submissions, reflecting the global interests. After a rigorous peer-review process, we accepted six high-quality articles that address critical gaps, provided actionable insights, and reviewed advances in specific fields. By 5 June 2025, the topic has seen a total number of views of 9,138, a total article view count of 6,398, and a total number of downloads of 1,298. The accepted articles described microbial communities in systems for treating sewage sludge, food wastes, and wastewater, highlighting their roles and applications in biodegradation and resource recovery (Mendoza et al.; Bird et al.), solid waste treatment and management (Mironov et al.; Chen, Jiang, Zhao, et al.), and wastewater treatment (Li et al.; Chen, Jiang, Yang, et al.).

These studies demonstrated significant progress in developing microbial-based solutions for key environmental challenges. Mendoza et al. comprehensively reviewed biodegradation strategies for agricultural plastic waste, identifying bacteria, fungi, algae, and insect larvae as effective agents for low-density polyethylene degradation while outlining optimization parameters for enhanced efficiency. Bird et al. advanced microbial fuel cell technology by elucidating how substrate complexity and mass transfer conditions influence electroactive microbial communities (*Geobacteraceae, Rhodocyclaceae*, and *Burkholderiaceae*), providing crucial insights for coupling MFCs with anaerobic digestion systems.

In organic waste management, Mironov et al. developed an innovative composting approach by inoculating *Bacillus* and *Penicillium* strains, demonstrating how microbial composition and timing regulate metabolic pathways for accelerated food waste degradation (Mironov et al.). Another review by Chen, Jiang, Zhao, et al. highlighted cutting-edge biotechnologies for in situ sludge reduction, including enzymatic hydrolysis, phage therapy, and biofilm manipulation, presenting sustainable alternatives to conventional sludge disposal methods.

To combat antibiotic resistance and remove contaminants in wastewater, Li et al. isolated multidrug-resistant bacteria (*Microbacterium, Chryseobacterium, Lactococcus lactis*, and *Psychrobacterin* strains) from wastewater effluents and evaluated natural compounds like curcumin and emodin as potential control agents. For saline wastewater treatment, Chen, Jiang, Yang, et al. characterized the resilience of *Thiobacillus*-dominated communities in sulfur-based denitrification systems under high sulfate stress, offering practical solutions for industrial wastewater applications.

While studies in this topic have significantly advanced our understanding of microbial communities in environmental engineering systems, several critical research gaps remain, which warrants further investigation: (1) mechanistic understanding of microbial community dynamics in engineered environments; (2) improved characterization of structure-function relationships in complex microbial systems; (3) systematic evaluation of microbial community management strategies and investigation of long-term stability and resilience of engineered microbiomes; (4) how to address the scaling challenges from laboratory to full-scale implementation.

